# Werner helicase interacting protein 1 contributes to G-quadruplex processing in human cells

**DOI:** 10.1038/s41598-024-66425-y

**Published:** 2024-07-08

**Authors:** Lili Hegedus, Agnes Toth, Gabor M. Harami, Janos Palinkas, Nargis Karatayeva, Eniko Sajben-Nagy, Szabolcs Bene, Sara Afzali Jaktajdinani, Mihaly Kovacs, Szilvia Juhasz, Peter Burkovics

**Affiliations:** 1 Institute of Genetics, Biological Research Centre, HUN-REN Szeged, Szeged, Hungary; 2 Doctoral School of Biology, Faculty of Science and Informatics, University of Szeged, Szeged, Hungary; 3 ELTE-MTA Momentum Motor Enzymology Research Group, Department of Biochemistry, Eötvös Loránd University, Budapest, Hungary; 4 HUN-REN-ELTE Motor Pharmacology Research Group, Department of Biochemistry, Eötvös Loránd University, Budapest, Hungary; 5 HCEMM Cancer Microbiome Core Group, Szeged, Hungary; 6 Institute of Biochemistry, Biological Research Centre, HUN-REN Szeged, Szeged, Hungary; 7 Present Address: Laboratory of Single Molecule Biophysics, National Heart, Lung and Blood Institute, National Institutes of Health, Bethesda, MD 20892 USA

**Keywords:** WRNIP1, G-quadruplex, PIF1, DNA helicase, Genome stability, Replication, DNA, Biochemistry, DNA-binding proteins, DNA damage and repair

## Abstract

Genome replication is frequently impeded by highly stable DNA secondary structures, including G-quadruplex (G4) DNA, that can hinder the progression of the replication fork. Human WRNIP1 (Werner helicase Interacting Protein 1) associates with various components of the replication machinery and plays a crucial role in genome maintenance processes. However, its detailed function is still not fully understood. Here we show that human WRNIP1 interacts with G4 structures and provide evidence for its contribution to G4 processing. The absence of WRNIP1 results in elevated levels of G4 structures, DNA damage and chromosome aberrations following treatment with PhenDC3, a G4-stabilizing ligand. Additionally, we establish a functional and physical relationship between WRNIP1 and the PIF1 helicase in G4 processing. In summary, our results suggest that WRNIP1 aids genome replication and maintenance by regulating G4 processing and this activity relies on Pif1 DNA helicase.

## Introduction

The faithful replication of the genetic material is essential for normal cell function and genome integrity. Altered DNA bases or DNA structures can block the replication fork generating mutations that can accumulate through time and cause carcinogenesis^1^.

G-quadruplex (G4) structures can be formed on guanine-rich single-stranded DNA or RNA regions by Hoogsteen base pairing. Their cellular importance is demonstrated by the high incidence of G4-forming regions in the human genome and their involvement in several basic cellular processes, such as telomere protection, IgG recombination, replication origin firing, and regulation of gene expression^2–4^. G4s may form an impediment for DNA replication machinery, consequently, proper replication of G4-forming sequences is crucial^5,6^. However, the mechanism and regulation of G4 resolution and replication is poorly understood. Several human G4-unwinding DNA helicases were described, which may contribute to the unwinding of G4 structures during replication^7^. In the absence of these helicases, the formed G4-quadruplexes can inhibit the movement of the replication machinery.

PIF1 is an evolutionary conserved 5’-3’ DNA helicase functioning both in the mitochondria and nucleus and has been implicated in G4 processing^8,9^. The *S. cerevisiae* Pif1 has well-described nuclear functions^10^, it is involved in different replication processes^11–15^ and indispensable in G4 maintenance^16–20^. Unlike the yeast Pif1, our knowledge about the human PIF1 is incomplete. Even though its G4-unwinding property has been thoroughly analysed in vitro^21^, PIF1’s in vivo function in human cells is not clear yet. Although the human PIF1 doesn’t seem to be as essential as yeast Pif1 in the replication of G4-prone regions, its involvement in breast cancer development underlines its importance in genome maintenance^11,22^.

The RecQ family of DNA helicases is a large, conserved group from bacteria to humans^23^. In humans two members of this group, the BLM and WRN helicases, can unwind G4 structures in 3’-5’ direction in ATP-dependent (WRN) and independent (BLM) manner^24^. Although the *in vivo* action of these DNA helicases on G4 DNA is very important, the regulation of their in vivo selection has not been explored yet^24–27^.

The human Werner Interacting Protein 1 (WRNIP1) is an evolutionarily conserved AAA^+^ family ATPase that was identified in a yeast two-hybrid screen as an interacting partner of the WRN helicase^28^. It is homologous to the *Escherichia coli* RarA (also known as MgsA)^29^ and yeast Mgs1 proteins^28^. WRNIP1 interacts with several proteins involved in DNA replication and repair processes^30–35^ and can stabilize stalled replication forks^32,33^. WRNIP1 has an important connection with the RAD6/RAD18 ubiquitin ligase enzyme complex, which complex is the major regulator of the function of the DNA damage bypass by translesion synthesis DNA polymerases^36^. Simultaneous deletion of yeast *mgs1* and *rad6* shows a synthetically lethal phenotype^37^, while in vertebrates *WRNIP1*^-/-/-^/*RAD18*^-/-^ double-knockout DT40 cells (*WRNIP1* gene is triploid in DT40 cells) are viable with a reduced growth-rate indicating partially different protein functions in yeast and vertebrates^38^. The amount of sister-chromatid exchange (SCE) events in *WRN*^-/-^/*WRNIP1*^-/-/-^ double-mutant DT40 cells was partially additional, which indicates a WRN-independent role of WRNIP1 in the absence of exogenous DNA damage^39^. Similar effect was also observed in *BLM*^-/-^/*WRNIP1*^-/-/-^ knock-down chicken cells^40^. These results indicate a RecQ-helicase-independent function of WRNIP1, potentially in DNA replication, because no exogenous damage was needed for these phenotypes. In addition, recently WRNIP1 was identified as a crucial factor in counteracting transcription-associated DNA damage, potentially by facilitating R-loop disassembly^41^.

In this study, we provide insight into the function of human WRNIP1 in G4 resolution. We show that WRNIP1 binds to G4 DNA oligonucleotides with high affinity *in vitro*. Depletion of WRNIP1 in human cells leads to an increased abundance of G4 structures accompanied by impaired DNA replication upon PhenDC3-treatment, ultimately leading to a higher frequency of chromosome abnormalities and genome instability. Furthermore, we show that WRNIP1 and PIF1 can localize to the same protein complex. Taken together, our results suggest that WRNIP1 is involved in G4 metabolisms in concert with the PIF1 DNA helicase.

## Results

### WRNIP1 has a robust G4 DNA binding affinity

**Figure 1:**
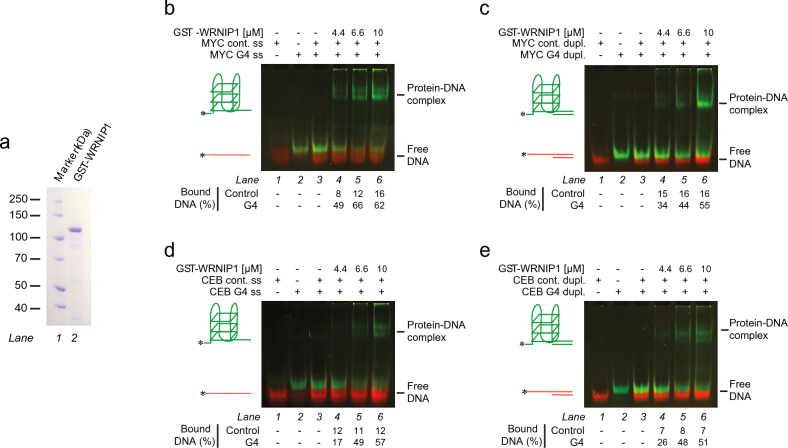
WRNIP1 protein preferentially binds G4 structures. (**a**) Purified GST-WRNIP1 protein. (**b**-**c**). Competitive EMSA experiment with (**b**) single-stranded or (**c**) partial duplex 5’-FITC labelled MYC G4 (green) and 5’-Cy3 labelled MYC control (red) substrates. (**d**-**e**). Competitive EMSA experiment with (**d**) single-stranded or (**e**) partial duplex 5’-FITC labelled CEB G4 (green) and 5’-Cy3 labelled CEB control (red) substrates. The percentage of DNA substrate in protein-DNA complex is indicates in % below the corresponding lanes.

After purification of the human GST-WRNIP1 fusion protein (Fig. 1A, referred as WRNIP1 in the text), we tested its G4 DNA-binding affinity towards two G4-forming ssDNA molecules containing either the human c-MYC oncogene promoter sequence (Myc2345) or the human CEB minisatellite region (CEB25) (Supplementary Table 1). Both regions are well-characterized G4-forming sequences under our experimental conditions^42,43^.

Both ssDNA molecules (referred as MYC G4 ss and CEB G4 ss) were 5’ labelled with a FITC fluorophore; the substrates contained a G4-forming sequence followed by an additional 21-nucleotide-long region that does not form a specific secondary structure. As controls, we used 5’ Cy3-labelled variants of the aforementioned DNA molecules, in which G4-forming guanines were changed to other nucleotides to avoid G4-formation (MYC cont. ss and CEB cont. ss) (Supplementary Table 1). Indeed, in competitive gel shift experiments, WRNIP1 preferentially bound both G4-forming molecules as compared to the negative controls (Fig. 1B and D). Previously WRNIP1 was shown to interact with replication forks and replication fork proteins^44^. Therefore, we tested its binding to DNA structures mimicking the primer-template junction of the replication fork with or without a G4 structure, by hybridizing a complementary unlabelled oligonucleotide to the 3’ single-stranded region of previously used G4-forming and control DNA molecules. WRNIP1 preferentially bound the G4-containing partial duplex substrates compared to their control pairs (Fig. 1C and E), similarly to that observed for the single-stranded variants.

To precisely determine the differences in the binding efficiency towards the G4 and control substrates, fluorescence anisotropy experiments were performed with 5’ fluorescein labelled DNA molecules. Besides the previously described MYC, CEB and control DNA-molecules, we tested binding to a GC-rich ssDNA molecule with equal GC-content to the MYC G4; and a C-rich substrate, which is the complementary strand of the MYC G4 sequence. The measured binding affinities were significantly higher for G4 substrates compared to their control pairs in every case (Table 1). Binding was the strongest towards the single-stranded CEB G4 molecule (*K*_d_ = 65 ± 6 nM), and it was 2.8-times weaker for the single-stranded MYC G4 indicating that differences in the G4 structure may finetune the binding affinity. In addition, the moderate differences between the binding efficiencies of the single-stranded and partial duplex molecules show that not only the G4 structure but the adjacent regions are also involved in the binding (Supplementary Fig. 1A–F, Table 1). However, the similarly weak binding affinity to the single-stranded or partial duplex control, GC-rich and C-rich molecules further underlines that binding is specific to G4 structures and is much stronger than binding to ssDNA, dsDNA or the ss-dsDNA junction.

**Table 1:** *K*_d_ and Hill-coefficient values determined from Supplementary Fig. 1.

Substrate			*K*_d_ ± fitting error (nM)	N (Hill-coefficient) ± fitting error	Number of biological replicates
MYC single-stranded	G4		184 ± 36	0.55 ± 0.053	3
	Control		> 5000	N/A	3
	GC-rich control		> 5000	N/A	3
	C-rich control		3496 ± 511	0.96 ± 0.176	3
MYC partial duplex	G4		108 ± 45	0.88 ± 0.16	3
	Control		> 5000	N/A	3
CEB single-stranded	G4		65 ± 6	0.95 ± 0.12	3
	Control		> 5000	N/A	3
CEB partial duplex	G4		221 ± 44	1.1 ± 0.15	3
	Control		> 5000	N/A	3
MYC single-stranded	ATP-	G4	175 ± 43	0.72 ± 0.11	3
		Control	> 3000	N/A	3
	ATP +	G4	183 ± 52	0.66 ± 0.10	3
		Control	> 3000	N/A	3

It has been suggested that WRNIP1’s ATP-binding could affect its DNA-binding affinity. However, we found that binding to the single-stranded MYC G4 and MYC control molecules is not influenced by the presence of 1 mM ATP (Supplementary Fig. 1 G-H, Table 1), indicating that the ATP-binding by WRNIP1 is not linked to G4 structure recognition.

Interestingly, WRNIP1 binding to the single-stranded MYC G4 structure is moderately negatively cooperative both in the absence or presence of ATP, based on the analyses of the binding isotherms using the Hill-equation. This may indicate formation of protein complexes on this DNA molecule; however for the other analysed DNA structures cooperativity was not observed (Table 1), Hill-coefficients were close to 1. Previously it was also reported that the WRNIP1 protein could form a homo-octamer complex; however inference for such specific quaternary structures cannot be made from our DNA-binding results. Nevertheless, the gel shift and anisotropy experiments together underline that WRNIP1 binds to G4 structures with high affinity and specificity.

### WRNIP1 participates in the replication of G4-forming sequences

**Figure 2:**
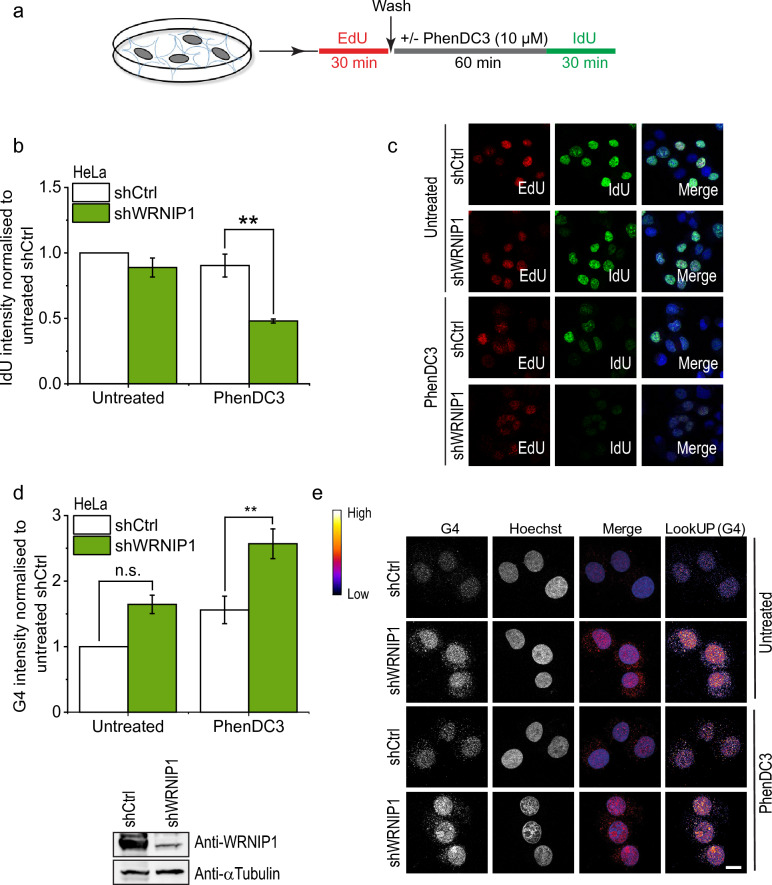
WRNIP1 participates in the replication of G4-forming sequences. (**a**) Schematic representation of the assay for the visualization of DNA replication. HeLa cells were pulse labelled with EdU for 30 min, washed out and then treated or not treated with G4 ligand PhenDC3 (10 µM) for 1h. IdU was added for 30 min, then IdU signal was detected by anti-IdU antibody and EdU was detected with click-it reaction, as described in the Materials and methods section. (**b**) Quantification of IdU intensity in shCtrl and shWRNIP1 carrying HeLa cells. Where indicated, cells were treated with G4 ligand PhenDC3 (10 µM). IdU signal intensity was measured as indicated in (**a**). IdU intensity was measured in 100 EdU positive nuclei per sample. (**c**) Representative images from (**b**). Scale bars, 20 μm. (**d**) Quantification of G4 intensity in shCtrl and shWRNIP1 carrying HeLa cells. Where indicated, cells were treated with G4 ligand PhenDC3 (10 µM) for 1 h. Cells were collected after 6 h following PhenDC3-tratement. G4 intensity was measured in 100 nuclei per sample. (**e**) Representative images from (**d**). Scale bars, 10 μm. The depletion of WRNIP1 was tested by Western blot analysis (bottom). Error bars represent SEM of 3 biological replicates on graphs (**b**) and (**d**). p-values were obtained by ANOVA (Origin Pro) (*P < 0.05, **P < 0.01, and ***P < 0.001). Statistical data is provided in Table S4. Original Western blot membrane is showed in Supplementary Fig. 5.

Based on the robust and specific G4-binding by WRNIP1 observed in vitro, we hypothesized that WRNIP1 may contribute to the replication of G4-prone sequences in vivo. Therefore, we assessed cell replication fork dynamics after G4 structure stabilization with PhenDC3-treatment using a previously described method^45^. Briefly, replication foci were pulse-labelled using EdU (5-ethynyl-2-deoxyuridine) in control and WRNIP1-depleted HeLa cells before treatment. Cells were treated then with PhenDC3 or as a control with DMSO, and newly replicated regions were labelled with IdU (5-iodo-2’-deoxyuridine) nucleotide analogue (Fig. 2A). Control experiments, in which a high UV dose (100 J/m^2^) was applied instead of PhenDC3, confirmed that our applied methodology can reliably report replication defects. Upon UV-treatment we detected a strong reduction of IdU signal intensity in EdU-labelled cells (Supplementary Fig. 2A). Silencing of WRNIP1 (shWRNIP1) without PhenDC3-treatement did not result a significantly different IdU-incorporation level as compared to the control cells (shCtrl) (Fig. 2B and C and Supplementary Fig. 3). In addition, PhenDC3-treatment, despite its G4-stabizing effect, did not affect the overall progression of DNA synthesis in control cells. Importantly, depletion of WRNIP1 resulted in a significant decrease in DNA synthesis progression after PhenDC3-treatment (Fig. 2B and C). This finding strongly indicates that WRNIP1 has a role in the replication of G4-prone DNA regions.

To visualize the amount of G4 structures in HeLa cells, we performed immunostaining using a G4-specific antibody. As expected, PhenDC3-treatment in control cells increased the intensity of G4-staining (Fig. 2D and E), highlighting both the G4 stabilization effect of PhenDC3 and the usability of the antibody. WRNIP1-silencing alone slightly increased the amount of formed G4 structures without PhenDC3-treatment and this amount was significantly increased after PhenDC3-treatment. This observation indicates that WRNIP1 has an important function in the resolution of G4 structures.

**Figure 3:**
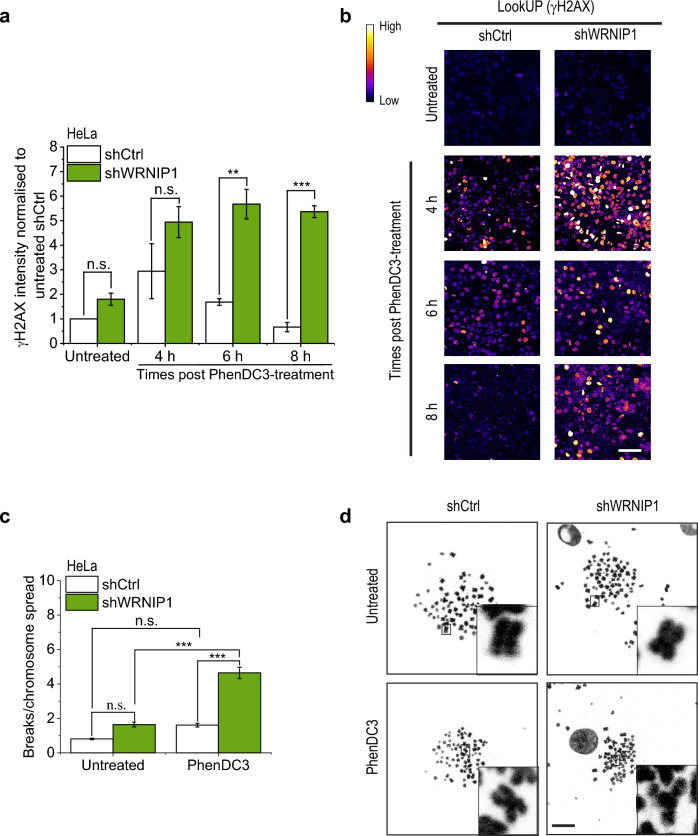
Depletion of WRNIP1 increases the frequency of chromosome breaks upon PhenDC3 treatment. (**a**) Quantification of γH2AX intensity in shCtrl and shWRNIP1 carrying HeLa cells. Where indicated, cells were treated with G4 ligand PhenDC3 (10 µM). Cells were immunostained at different time points after PhDC3-treatment. γH2AX intensity was measured in 100 nuclei per sample. (**b**) Representative images of untreated and PhenDC3-treated cells from (**a**). Scale bars, 50 μm. (**c**) Chromosome aberration assay. Quantification of chromosome breaks per chromosome spread in shCtrl and shWRNIP1HeLa cells. Where indicated, cells were treated with G4 ligand PhenDC3 (10 µM). After 12 h, samples were treated with colchicine for 6 h to arrest the cells in M phase. Chromosome aberration was counted in 40 chromosome spreads per sample. (**d**) Representative images from (**c**). Scale bars, 10 μm. Error bars represent SEM of 3 biological replicates on graphs (**B**) and (**D**). p-values were obtained by ANOVA (Origin Pro) (*P < 0.05, **P < 0.01, and ***P < 0.001). Statistical data is provided in Table S4.

Previously it was shown that the stabilized G-quadruplex can block the replication fork, which results in the formation of short ssDNA regions that can be converted into DSBs^46^. To determine the consequences of decreased G4-resolution and replication we measured the γH2AX signal intensity, which indicates the formation of double-strand breaks and the presence of long single-stranded DNA stretches^47–49^, after PhenDC3 treatment in control and WRNIP1-depleted cells. PhenDC3-treatment dramatically increased the γH2AX signal intensity in WRNIP1-depleted S-phase HeLa (Supplementary Fig. 2B) and HT1080 cells (Supplementary Fig. 2C) in contrast to the controls and in correlation with the observed higher amount of formed G4 structures. In addition, permanent PhenDC3-treatment of WRNIP1-silenced cells resulted in a persistently increased γH2AX signal compared to control cells (Fig. 3A and B). This result was corroborated by another G4-stabiliser, pyridostatin (Supplementary Fig. 4 A and B).

The increased γH2AX signal may indicate a higher DSB frequency, thus next we assessed how genome integrity is affected by the lack of WRNIP1. The number of chromosome breaks was counted in control and WRNIP1-silenced cells upon and without PhenDC3-treatment (Fig. 3C and D). We found that silencing of WRNIP1 in the absence of G4 stabilization, as well as PhenDC3-treatment of the control cell line slightly increased the number of chromosome breaks. Compared to these controls, PhenDC3-treatment in WRNIP1-silenced cells significantly increased the frequency of chromosome breaks, in line with the higher accumulation of DNA lesions reported by the γH2AX signal (Fig. 3A and B). Taken together, the depletion of WRNIP1 has significant impact on G4 processing, because its depletion resulted in reduced DNA synthesis, prolonged G-quadruplex lifetime, increased γH2AX signal and increased number of chromosome breaks.

### WRNIP1 and PIF1 cooperates to resolve G-quadruplexes

Previously we suggested the possible interaction between the Mgs1 and Pif1 in yeast cells^50^. Therefore, we tested this between the human WRNIP1 and human PIF1 utilizing a previously described protein fragment complementation assay involving the split VENUS reporter plasmid system^51^. In this assay the N- and C-terminal fragments of the fluorescent VENUS protein are expressed in fusion to proteins of interest separately. Upon close spatial localization of the chimeric proteins the N- and C-terminal VENUS fragments can assemble to form a fluorescent complex, while the separated fragments are not fluorescent.

**Figure 4:**
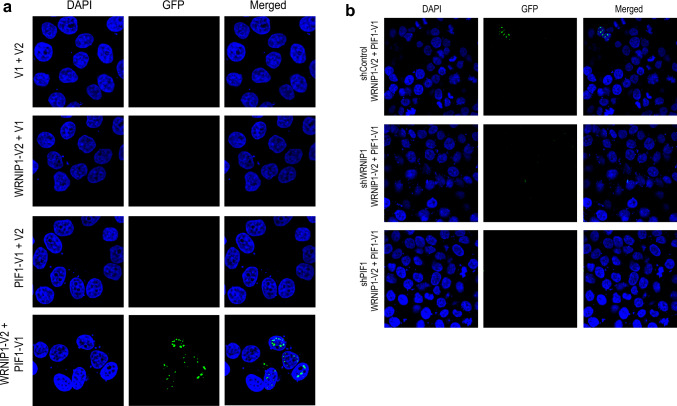
WRNIP1 interacts with PIF1 DNA helicase in vivo. (**a**) In a protein fragment complementation assay, we used the split Venus reporter plasmid system. PIF1 was cloned in fusion with the N-terminal part, and WRNIP1 was cloned in fusion to the C terminal part of Venus protein. Only the co-transfection of V1-PIF1 and V2-WRNIP1 resulted in green fluorescence sign, demonstrating the association of these two proteins and the specificity of the experiment. (**b**) The co-transfection of V1-PIF1, V2-WRNIP1, and WRNIP1 shDNS, or PIF1 shDNA, as indicated is resulted in the disappearing of the green fluorescence sign, proving the specific interaction between WRNIP1 and PIF1.

Upon simultaneous expression of PIF1 and WRNIP1 fused to the complementary N- and C-terminal fragments of the split VENUS fluorescent protein in HeLa cells, we observed the formation of multiple fluorescent foci (Fig. 4) in fluorescent microscopy experiments. Interestingly, the observed foci were relatively large and localized to regions with low DAPI staining, including the nucleoli. In control experiments, where VENUS fragments were expressed without fusion to PIF1 or WRNIP1 or when only either a fragment fused WRNIP1 or PIF1 was expressed together with the complementary, non-fused VENUS fragment foci were not observed. Furthermore, the silencing of either WRNIP1 or PIF1 also resulted in the loss of foci, thereby further corroborating the specificity of the observed result. These results indicate that PIF1 and WRNIP1 exist as members of the same protein complex either due to direct physical interaction or due to a common binding partner.

**Figure 5:**
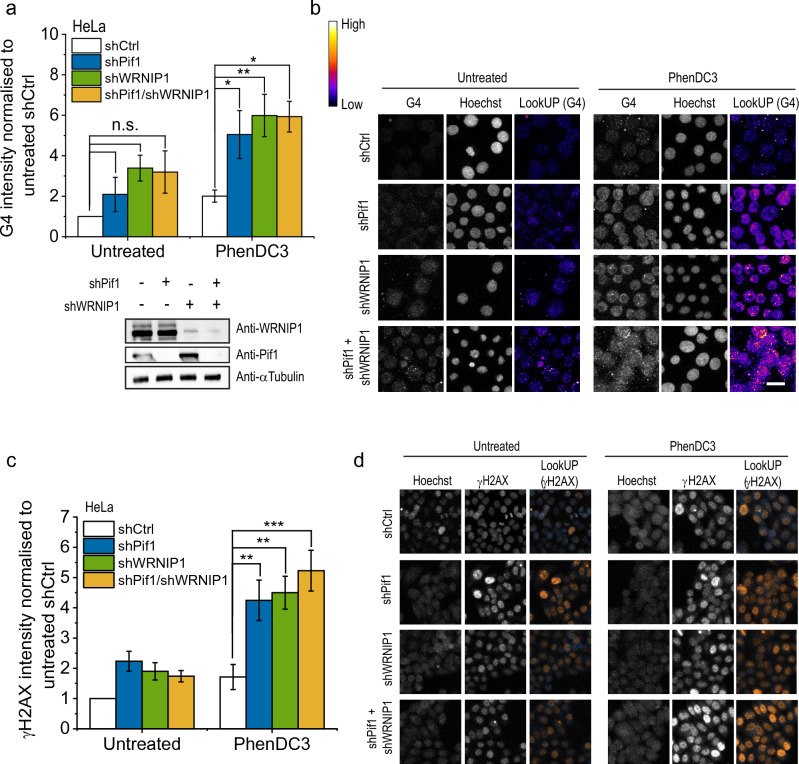
WRNIP1 cooperates with PIF1 in replication of G4 forming regions. (**a**). Quantification of G4 intensity in shCtrl, shWRNIP1, shPif1 and shPif1/shWRNIP1 HeLa cells. Where indicated, cells were treated with G4 ligand PhenDC3 (10 µM). Cells were collected and G4 intensity was measured in 100 nuclei per sample. The depletion of WRNIP1, Pif1 and Pif1/WRNIP1 was tested by Western blot (bottom). (**b**) Representative images from (**a**). Scale bars, 20 μm. (**c**) Quantification of γH2AX intensity in shCtrl, shWRNIP1, shPif1 and shPif1/shWRNIP1 HeLa cells. Where indicated, cells were treated with G4 ligand PhenDC3 (10 µM). Cells were collected and γH2AX intensity was measured in 100 nuclei per sample. (**d**) Representative images from (**c**). Scale bars, 20 μm. Error bars represent SEM of 3 biological replicates on graphs. p-values were obtained by ANOVA (Origin Pro) (*P < 0.05, **P < 0.01, and ***P < 0.001). Statistical data is provided in **Table S4**. Original Western blot membrane is showed in Supplementary Fig. 5.

In line with the notion that PIF1 and WRNIP1 may function together, the simultaneous and separate depletion of WRNIP1 and PIF1 in HeLa cells increased the intensity of G4-staining (Fig. 5A, B) and γH2AX-staining (Fig. 5C, D) to comparable levels. In summary, we demonstrated here the physical and functional interaction of human WRNIP1 and PIF1 proteins.

## Discussion

Stable secondary structures forming on single stranded nucleic acids, such as G4 quadruplexes, can induce replication fork pausing which can lead to the formation of DNA lesions, including DSBs. Thus, persistent G4 structures have been proposed to impact genome stability and gene function by interfering with replication fork progression, transcription elongation, and DNA repair mechanisms^5,52–54^. Several studies demonstrated a strong association between G4 structures and human diseases^55,56^, indicating a strict and precise control over G4 homeostasis in healthy cells. G4-unwinding DNA helicases are key effectors in the process^7,57,58^; however the regulation of these enzymes and their interplay with the replication machinery is not well understood.

The observed robust, specific, and ATP-independent G4-binding of WRNIP1 and the protein`s previously described tight connection with the replication complex and DNA helicases^30,33,35,38–40,44,59–63^ suggests the possibility of WRNIP1 contribution to G4 replication and homeostasis. Indeed, in line with this idea we detected a higher level of persistent G4s in the absence of WRNIP1 *in vivo*, which was further elevated by PhenDC3 mediated stabilization of G4s. Notably, PhenDC3-treatement also slowed down the rate of DNA replication, showing a more pronounced effect in WRNIP1-depleted cells, and the slower replication rate and increased persistence of G4 structures upon PhenDC3-treatment correlated with an increased γH2AX signal intensity. These results indicate that G4 structures persisting in the absence of WRNIP1 activity may lead to the formation of DNA lesions, likely DSBs, due to replication stalling or impended DNA repair. The increased DSB frequency can lead to genomic instability and chromosome aberrations and accordingly we observed a significantly higher chromosome aberration frequency in WRNIP1-silenced PhenDC3-treated cells. Recently it has been shown that WRNIP1 limits the formation of transcription-coupled R-loops^41^. R-loops can facilitate formation of G4 structures on the noncoding template DNA strand^64,65^ which, in turn, can influence the stability of R-loops. Therefore, it is possible that WRNIP1 regulates the R-loop formation by modifying the lifetime of these G4 structures. In line with our findings, the negative effect of WRNIP1-depletion on genome stability was also observed previously^32^; however a link between WRNIP1 activity and G4 homeostasis has not been identified.

Recently we demonstrated the functional relationship between the yeast Mgs1 and yeast Pif1 helicase in G4 processing^50^. Motivated by this observation, we tested the interplay between WRNIP1 and human PIF1. Silencing of PIF1 resulted in elevated G4 and γH2AX signal intensities in HeLa cells confirming its previously proposed contribution to G4 processing pathways^16,21^. Importantly, simultaneous-depletion of WRNIP1 and PIF1 resulted in a similar increase in G4 and γH2AX signal intensity as their independent depletion. This finding highlights the functional cooperation of WRNIP1 and PIF1 is G4 processing, supported by the results of our fragment complementation-based assay, which suggests the direct association of these two proteins.

In summary, our study reveals that WRNIP1 plays an important role in G4 processing mechanisms during DNA replication likely by orchestrating the coordinated action of replicative proteins and the G4 unwinding protein PIF1.

## Methods

### Generation of plasmid constructs

The cDNA encoding full-length human WRNIP1 protein was cloned into the pENTR4 Gateway entry vector (*Thermo Fischer*) using NcoI and EcoRI restriction enzymes (*Thermo Fischer*), resulting the pBP378 plasmid. For WRNIP1 protein expression the WRNIP1 cDNA was cloned in fusion with an N-terminal glutathione S-transferase (GST) tag under the control of the *S. cerevisiae* galactose-inducible phosphoglycerate promoter using the Gateway cloning system (*Invitrogen*), which resulted pBP069 plasmid.

For protein fragment complementation assay, we cloned first the PIF1 cDNA (Origene RC223376) to the pENTR2B plasmid, resulted in pBP463. We cloned the PIF1 and WRNIP1 cDNA in fusion with N-terminal (V1) or C-terminal (V2) fragment of VENUS YFP^51^, resulting the pBP484 and pBP483 plasmids, respectively.

### Protein expression and purification

The WRNIP1 protein was overexpressed in protease-deficient BJ5464 yeast strain. Cells were grown to stationer phase in synthetic medium lacking leucine to select for cells transformed with the used expression vector. The culture was diluted tenfold in fresh medium lacking dextrose, but containing 2% w/v lactic acid and 3% v/v glycerol. This was followed by overnight incubation with addition of galactose to 0.2% w/v final concentration. Cells were harvested and disrupted after a 10 h incubation at 30 °C in buffer P (20 mM Tris–HCl pH 7.5, 1 mM dithiothreitol (DTT), 0.01% v/v Nonidet P-40, 10% v/v glycerol) supplemented with 1 M NaCl, 5 mM EDTA, and protease inhibitor mixture (*Roche Applied Science*). After centrifugation, the supernatant of the cell extract was loaded onto glutathione-Sepharose columns (*GE Healthcare*). First, the column was washed with buffer P + 1 M NaCl followed by washing with buffer P + 500 mM KCl. The GST-WRNIP1 protein was eluted with 20 mM reduced glutathione in buffer P supplemented with 500 mM KCl. WRNIP1-containing fractions were concentrated on Microcon-30 column (*Merck Millipore*), frozen in liquid nitrogen, and stored at − 80 °C.

### Preparation of DNA molecules

DNA substrates (Supplementary Table 1) used for gel shift assay and fluorescent anisotropy measurements were prepared by heating the indicated oligonucleotides in buffer A (25 mM Tris–HCl pH 7.5, 50 mM KCl) for 5 min at 100 °C and cooling them slowly overnight.

### Gel shift assay

Different concentrations of purified GST-WRNIP1 (as indicated in the figures) were incubated in buffer C (50 mM Tris–HCl pH 7.5, 150 mM KCl) with 50 nM fluorescein- and/or Cy3-labelled DNA substrate for 15 min at 25 °C. Samples were run on native polyacrylamide gel (6% w/v) in 0.5 X TB buffer (45 mM Tris–borate) and imaged using a Typhoon Trio Imager (GE Healthcare). The binding efficiency was calculated as the ratio of the total intensity of protein bound DNA species and the total intensity of free plus bound DNA species, and measured with ImageJ software.

### Fluorescence anisotropy

Fluorescein(FITC)-labelled DNA substrates (10 nM) were incubated with increasing amount of purified GST-WRNIP1 in buffer D (50 mM Tris–HCl pH 7.5, 90 mM KCl, 5 mM EDTA with or without 1 mM ATP and 1.25 mM MgCl_2_) at 25 °C. Fluorescence anisotropy was measured in Synergy H4 Hybrid Multi-Mode microplate reader (*BioTek*). The indicated *K*_d_ values were calculated by Origin 8.0 software using the Hill-equation.

### Cell lines and cell culture

HeLa and HT1080 cells were cultured in DMEM (*Sigma*) supplemented with 10% v/v FBS, 100 μg/mL penicillin, 100 U/mL streptomycin and 1% v/v NEAA and maintained at 37 °C in a 5% CO_2_ incubator. Cells were transfected with plasmids using Dharmafect Duo (*Dharmacon*) or PEI (*Sigma*) according to manufacturer’s instructions. Experiments were performed 48 h after plasmid and/or shDNA transfection (Supplementary Table 2). The downregulation of the indicated genes was verified by western blot using specific antibodies (Supplementary Table 3).

### Immunofluorescence

Cells were washed once with PBS and fixed with 3% w/v PFA for 10 min. Cells were then permeabilized with 0.5% v/v Triton X-100 in PBS for 10 min, washed with PBS and incubated in blocking buffer (3% BSA, 0.1% v/v Triton X-100 in PBS) for 1 h at room temperature. Samples were incubated in primary antibodies diluted in blocking buffer overnight at 4 °C. Cells were washed three times with 0.1% v/v Triton X-100 in PBS and incubated with fluorescently-tagged secondary antibodies diluted in blocking buffer at room temperature for 1 h in the dark. Cells were washed twice with 0.1% v/v Triton X-100 in PBS and counterstained with Hoechst (1 μg/mL in PBS) for 10 min.

For G4 staining cells were collected in tube and washed with PBS, then treated with 75 mM KCl for 30 min at 37 °C. Cells were collected with centrifugation and the cell pellet was resuspended in 75 mM KCl. The isolated nuclei were concentrated with cytospin onto microscope coverslips and fixed with 3% w/v PFA for 10 min. Nuclei were then permeabilized with 0.5% v/v Triton X-100 in PBS for 10 min, washed three times with PBS and incubated in blocking buffer (3% w/v BSA, 0.1% v/v Triton X-100 in PBS) for 1 h at room temperature. Samples were incubated with anti-G4 antibody (*Sigma,* BG4) diluted in blocking buffer for 4 h at 4 °C, then in anti-FLAG antibody (*Sigma*) overnight at 4 °C. Cells were washed three times with 0.1% v/v Triton X-100 in PBS before incubation with fluorescently-tagged secondary antibody/antibodies diluted in blocking buffer at room temperature for 1 h in the dark. Cells were washed twice with 0.1% v/v Triton X-100 in PBS and counterstained with Hoechst (1 μg/mL in PBS) for 10 min.

Z-stacks of images were acquired on a LSM800 confocal setup with a Plan-Apochromat 20x/0.8 M27 or a Plan-Apochromat 63x/1.4 Oil DIC M27 objective, and controlled by the ZEN 2.3 software^45^. The raw images were analysed in Fiji (ImageJ) after generating the maximum intensity projections of z-stacks. The Hoechst stained DNA was used to segment the nuclei, the mean WRNIP1-signal and PIF1-signal intensities of 100 nuclei per experiment are plotted normalized to the untreated control^45^.

### EdU and IdU incorporation assay

The protocol was carried out as previously described^45^. HeLa cells were labelled with 10 µM EdU (5-ethynyl-2'-deoxyuridine, *Baseclick*, BCK-EdU555) for 30 min and washed with fresh culturing medium. Cells were then treated or not treated with 10 µM PhenDC3 for 1 h and labelled with 200 µM IdU (5-Iodo-2’-deoxyuridine, Sigma) thymidine analog for 30 min. Cells were fixed with 3% w/v PFA for 10 min and treated with 2.5 M HCl for 30 min. Cells were washed three times with PBS, then immunostained with anti-IdU primary antibody and fluorescently tagged secondary antibody. EdU-signal was visualized by a Click-IT Kit (*Baseclick*) according to the manufacturer’s instructions and nuclei were stained with Hoechst (1 μg/mL in PBS) for 10 min. The signal of EdU and IdU foci were imaged with a LSM800 confocal setup with a Plan-Apochromat 20x/0.8 M27 or a Plan-Apochromat 63x/1.4 Oil DIC M27 objective, and controlled by the ZEN 2.3 software. Fluorescence excitation was performed using diode lasers at 405, 488 and 561 nm. The raw images were analyzed in Fiji (ImageJ) after generating the maximum intensity projections of z-stacks^45^. The mean IdU-signal intensity of 100 EdU positive cells per experiment is plotted normalized to the untreated control.

### γH2AX intensity assay

Cells were plated onto glass coverslips containing culture dishes. 24 h later the coverslips with different cell types were pulse-labelled or not pulse-labelled with EdU. Cells were then treated or not treated with 10 µM PhenDC3 (*Sigma*) or 10 µM Pyridostatin (*Sigma*) for various times, washed with fresh medium and incubated for different times. In another experiment, cells were treated with 10 µM PhenDC3 and incubated for 4 h or 6 h or 8 h. After the desired time, cells were fixed with 3% w/v PFA, immunostained with anti-γH2AX antibody, the EdU-signal and nuclei were visualized with EdU Click-IT Kit and Hoechst (1 μg/mL in PBS) staining. The raw images were analysed in Fiji (*ImageJ*) after generating the maximum intensity projections of z-stacks^45^. The Hoechst stained DNA was used to segment the nuclei and the mean intensity of γH2AX-signal from 100 nuclei per experiment is plotted normalized to the untreated control.

### Chromosome aberration analysis

HeLa WT cells were transfected with shCtrl or shWRNIP1. After 40 h cells were treated with 10 µM PhenDC3 (*Sigma*) for 24 h, then 0.5 µg/mL colchicine (*Sigma*) and 20 mg/mL caffeine (*Sigma*) for 8 h to enrich mitotic cells. To obtain chromosome spreads, cells were collected, resuspended in 75 mM KCl for 30 min at 37 °C, centrifuged at 200 × g at 4 °C for 10 min, then fixed (3:1 methanol: acetic acid) 3 times. Chromosomes were spread onto cover slips, air-dried and stained with Hoechst (1 μg/mL in PBS) for 20 min. The chromosome spreads were captured on a LSM800 confocal setup and the number of chromosome aberrations per 40 chromosome spread was counted.

### Protein fragment complementation assay

HeLa cells were grown to confluency of about 90% in DMEM high glucose medium supplemented with 10% FBS prior to transfection. The indicated plasmids were transfected into the cells in 1:1 ratio. 48 h after the transfection cells were fixed on glass slides and images were acquired with the Olympus FV1000 confocal microscope.

### Data analysis

Means ± SEM values are reported in the paper, unless otherwise specified. Sample sizes (n) are given for the number of ensembled *in vitro* measurements performed using independent protein preparations (biological replicates, n = 3 unless otherwise specified). *In vivo* measurements were performed at least in 3 biological replicates. Data analysis was performed using Origin 8.0 (*OriginLab corp*) software and one-way ANOVA with Tukey's multiple comparison test. *p < 0.05; **p < 0.01; ***p < 0.001.

### Supplementary Information

Supplementary Information.

Supplementary Table 4.

## Data Availability

All data needed to evaluate the conclusions in the paper are present in the paper and/or the Supplementary Materials.

## References

[1] Aguilera A, García-Muse T (2013). Causes of genome instability. Annu. Rev. Genet..

[2] Bochman ML, Paeschke K, Zakian VA (2012). DNA secondary structures: Stability and function of G-quadruplex structures. Nat. Rev. Genet..

[3] Kamura T, Katsuda Y, Kitamura Y, Ihara T (2020). G-quadruplexes in mRNA: A key structure for biological function. Biochem. Biophys. Res. Commun..

[4] Lipps HJ, Rhodes D (2009). G-quadruplex structures: In vivo evidence and function. Trends Cell Biol..

[5] Lerner LK, Sale JE (2019). Replication of G quadruplex DNA. Genes.

[6] Wickramasinghe CM, Arzouk H, Frey A, Maiter A, Sale JE (2015). Contributions of the specialised DNA polymerases to replication of structured DNA. DNA Repair (Amst).

[7] Sauer M, Paeschke K (2017). G-quadruplex unwinding helicases and their function in vivo. Biochem. Soc. Trans..

[8] Lahaye A, Stahl H, Thines-Sempoux D, Foury F (1991). PIF1: A DNA helicase in yeast mitochondria. EMBO J..

[9] Schulz VP, Zakian VA (1994). The saccharomyces PIF1 DNA helicase inhibits telomere elongation and de novo telomere formation. Cell.

[10] Muellner J, Schmidt KH (2020). Yeast genome maintenance by the multifunctional PIF1 DNA helicase family. Genes.

[11] Buzovetsky O (2017). Role of the Pif1-PCNA complex in Pol δ-dependent strand displacement DNA synthesis and break-induced replication. Cell Rep..

[12] Deegan TD, Baxter J, Ortiz Bazán MÁ, Yeeles JTP, Labib KPM (2019). Pif1-family helicases support fork convergence during DNA replication termination in eukaryotes. Mol. Cell.

[13] Li S (2021). PIF1 helicase promotes break-induced replication in mammalian cells. EMBO J..

[14] Schauer GD (2020). Replisome bypass of a protein-based R-loop block by Pif1. Proc. Natl. Acad. Sci U.S.A..

[15] Sparks MA, Burgers PM, Galletto R (2020). Pif1, RPA, and FEN1 modulate the ability of DNA polymerase d to overcome protein barriers during DNA synthesis. J. Biol. Chem..

[16] Dahan D (2018). Pif1 is essential for efficient replisome progression through lagging strand G-quadruplex DNA secondary structures. Nucl. Acids Res..

[17] Lopes J (2011). G-quadruplex-induced instability during leading-strand replication. EMBO J..

[18] Paeschke K, Capra JA, Zakian VA (2011). DNA replication through G-quadruplex motifs is promoted by the saccharomyces cerevisiae Pif1 DNA helicase. Cell.

[19] Paeschke K (2013). Pif1 family helicases suppress genome instability at G-quadruplex motifs. Nature.

[20] Ribeyre C (2009). The yeast Pif1 helicase prevents genomic instability caused by G-quadruplex-forming CEB1 sequences in vivo. PLoS Genet..

[21] Sanders CM (2010). Human Pif1 helicase is a G-quadruplex DNA-binding protein with G-quadruplex DNA-unwinding activity. Biochem. J..

[22] Chisholm KM (2012). A genomewide screen for suppressors of Alu-mediated rearrangements reveals a role for PIF1. PLoS One.

[23] Bochman ML (2014). Roles of DNA helicases in the maintenance of genome integrity. Mol. Cell Oncol..

[24] Tippana R, Hwang H, Opresko PL, Bohr VA, Myong S (2016). Single-molecule imaging reveals a common mechanism shared by G-quadruplex-resolving helicases. Proc. Natl. Acad. Sci. U.S.A..

[25] Damerla RR (2012). Werner syndrome protein suppresses the formation of large deletions during the replication of human telomeric sequences. Cell Cycle.

[26] Drosopoulos WC, Kosiyatrakul ST, Schildkraut CL (2015). BLM helicase facilitates telomere replication during leading strand synthesis of telomeres. J. Cell Biol..

[27] Fry M, Loeb LA (1999). Human Werner syndrome DNA helicase unwinds tetrahelical structures of the fragile X syndrome repeat sequence d(CGG)(n). J. Biol. Chem..

[28] Kawabe YI (2001). A novel protein interacts with the werner’s syndrome gene product physically and functionally. J. Biol. Chem..

[29] Shibata T (2005). Functional overlap between RecA and MgsA (RarA) in the rescue of stalled replication forks in Escherichia coli. Genes Cells.

[30] Crosetto N (2008). Human Wrnip1 is localized in replication factories in a ubiquitin-binding zinc finger-dependent manner. J. Biol. Chem..

[31] Jiménez-Martín A (2020). The Mgs1/WRNIP1 ATPase is required to prevent a recombination salvage pathway at damaged replication forks. Sci. Adv..

[32] Leuzzi G, Marabitti V, Pichierri P, Franchitto A (2016). WRNIP 1 protects stalled forks from degradation and promotes fork restart after replication stress. EMBO J..

[33] Porebski B (2019). WRNIP1 protects reversed DNA replication forks from SLX4-dependent nucleolytic cleavage. iScience.

[34] Tsurimoto T, Shinozaki A, Yano M, Seki M, Enomoto T (2005). Human werner helicase interacting protein 1 (WRNIP1) functions as novel modulator for DNA polymerase δ. Genes Cells.

[35] Yoshimura A (2009). Physical and functional interaction between WRNIP1 and RAD18. Genes Genet. Syst..

[36] Hedglin M, Benkovic SJ (2015). Regulation of Rad6/Rad18 activity during DNA damage tolerance. Annu. Rev. Biophys..

[37] Hishida T, Ohno T, Iwasaki H, Shinagawa H (2002). Saccharomyces cerevisiae MGS1 is essential in strains deficient in the RAD6-dependent DNA damage tolerance pathway. EMBO J..

[38] Yoshimura A (2006). Functional relationships between Rad18 and WRNIP1 in vertebrate cells. Biol. Pharm. Bull..

[39] Kawabe Y (2006). Analyses of the interaction of WRNIP1 with Werner syndrome protein (WRN) in vitro and in the cell. DNA Repair (Amst).

[40] Hayashi T (2008). Vertebrate WRNIP1 and BLM are required for efficient maintenance of genome stability. Genes Genet. Syst..

[41] Valenzisi P, Marabitti V, Pichierri P, Franchitto A (2024). WRNIP1 prevents transcription-associated genomic instability. Elife.

[42] Eddy S (2014). Human Rev1 polymerase disrupts G-quadruplex DNA. Nucleic Acids Res..

[43] Amrane S (2012). Formation of pearl-necklace monomorphic G-quadruplexes in the human CEB25 minisatellite. J. Am. Chem. Soc..

[44] Yoshimura A, Seki M, Enomoto T (2017). The role of WRNIP1 in genome maintenance. Cell Cycle.

[45] Juhász S (2020). The chromatin remodeler ALC1 underlies resistance to PARP inhibitor treatment. Sci. Adv..

[46] Lemmens B, Van Schendel R, Tijsterman M (2015). Mutagenic consequences of a single G-quadruplex demonstrate mitotic inheritance of DNA replication fork barriers. Nat. Commun..

[47] Bonner WM (2008). γH2AX and cancer. Nat. Rev. Cancer.

[48] Redon CE (2010). Histone γH2AX and poly(ADP-ribose) as clinical pharmacodynamic biomarkers. Clin. Cancer Res..

[49] Nakamura AJ, Rao VA, Pommier Y, Bonner WM (2010). The complexity of phosphorylated H2AX foci formation and DNA repair assembly at DNA double-strand breaks. Cell Cycle.

[50] Zacheja T (2020). Mgs1 protein supports genome stability via recognition of G-quadruplex DNA structures. FASEB J..

[51] Hye YS (2007). Human tribbles-1 controls proliferation and chemotaxis of smooth muscle cells via MAPK signaling pathways. J. Biol. Chem..

[52] Estep KN, Butler TJ, Ding J, Brosh RM (2017). G4-Interacting DNA helicases and polymerases: Potential therapeutic targets. Curr. Med. Chem..

[53] van Kregten M, Tijsterman M (2014). The repair of G-quadruplex-induced DNA damage. Exp. Cell Res..

[54] Jackson SP, Bartek J (2009). The DNA-damage response in human biology and disease. Nature.

[55] Hanna R, Flamier A, Barabino A, Bernier G (2021). G-quadruplexes originating from evolutionary conserved L1 elements interfere with neuronal gene expression in Alzheimer’s disease. Nat. Commun..

[56] Wang E, Thombre R, Shah Y, Latanich R, Wang J (2021). G-Quadruplexes as pathogenic drivers in neurodegenerative disorders. Nucl. Acids Res..

[57] Castillo Bosch P (2014). FANCJ promotes DNA synthesis through G-quadruplex structures. EMBO J..

[58] Jimeno S (2018). The Helicase PIF1 facilitates resection over sequences prone to forming G4 structures. Cell Rep..

[59] Marabitti V (2020). Checkpoint defects elicit a WRNIP1-mediated response to counteract R-loop-associated genomic instability. Cancers (Basel).

[60] Leuzzi G, Marabitti V, Pichierri P, Franchitto A (2016). WRNIP1: A new guardian of genome integrity at stalled replication forks. Mol. Cell Oncol..

[61] Yoshimura A (2019). WRNIP1 controls the amount of PrimPol. Biol. Pharm. Bull..

[62] Socha A (2020). WRNIP1 Is recruited to DNA interstrand crosslinks and promotes repair. Cell Rep..

[63] Suzuki N (2016). A novel mode of ubiquitin recognition by the ubiquitin-binding zinc finger domain of WRNIP1. FEBS J..

[64] Lee CY (2020). R-loop induced G-quadruplex in non-template promotes transcription by successive R-loop formation. Nat. Commun..

[65] Miglietta G, Russo M, Capranico G (2021). G-quadruplex–R-loop interactions and the mechanism of anticancer G-quadruplex binders. Nucl. Acids Res.

