# Supplementary material for: Werner helicase interacting protein 1 contributes to G-quadruplex processing in human cells
Source: Sci Rep. 2024 Jul 8;14:15740. doi: 10.1038/s41598-024-66425-y (PMC11231340; doi:10.1038/s41598-024-66425-y)
Supplement: Supplementary file 1 — Supplementary Information. [file 41598_2024_66425_MOESM1_ESM.pdf]

Supplementary figure 1. WRNIP1 protein specifically binds to G4 structures *in vitro*.
(a-f). Fluorescence anisotropy measurement performed with (a) single-stranded MYC G4 (black) and MYC control (red), (b) partial duplex MYC G4 (black) and MYC control (red), (c) single-stranded MYC G4 (black) and MYC GC-rich control (green), (d) single-stranded MYC G4 (black) and MYC C-rich control (blue), (e) single stranded CEB G4 (black) and CEB control(red), (f) partial duplex CEB G4 (black) and CEB control (red). **(g-h).** Fluorescence anisotropy measurements performed with single-stranded (g) MYC control or (h) MYC G4 substrates in the presence (grey) or absence (blue) of ATP. Error bars represent SEM of 3 biological replicates. Solid lines represent best fits based on the Hill-equation. Fitted parameters are shown in **Table 1**.

Supplementary figure 2. WRNIP1 is involved in the replication of G4-forming sequences.
(a) Schematic representation of the assay for the visualization of the DNA replication. HeLa cells were pulse labelled with EdU for 30 minutes, washed out and then treated or not treated with UVC light 100 J/m^2. IdU was added for 30 minutes, then IdU signal was detected by anti-IdU antibody and EdU was detected with click-it reaction. UV treatment prevented IdU incorporation in the control experiment shown (representative images shown). Scale bars, 10 μm. **(b)** Quantification of γH2AX intensity in shCtrl and shWRNIP1 S-phase HeLa cells. Cells were pulse labelled with EdU (10 μM). Where indicated, cells were treated with G4 ligand PhenDC3 (10 μM). Cells were then immunostained at different time points after PhenDC3-treatment. γH2AX intensity was measured in 100 EdU positive nuclei per sample. The depletion of WRNIP1 was tested by Western blot analysis (**Figure 3**). **(c)** Quantification of γH2AX intensity in shCtrl and shWRNIP1 S-phase HT1080 cells. Cells were pulse labelled with EdU (10 μM). Where indicated, cells were treated with G4 ligand PhenDC3 (10 μM). Cells were then immunostained at different time points after PhenDC3-treatment. γH2AX intensity was measured in 100 EdU positive nuclei per sample. The depletion of WRNIP1 was tested by Western blot analysis (bottom). Error bars represent SEM of 3 biological replicates on graphs (b) and (c). p-values were obtained by ANOVA (Origin Pro) (*P<0.05, **P<0.01, and ***P<0.001). Statistical data is provided in **Table S4**. Original Western blot membrane is shown in **Supplementary Figure 5**.

Supplementary Figure 3. **WRNIP1 participates in the replication of G4-forming sequences.**
Representative microscope images from Figure 2 A and B.

Supplementary Figure 4. **Depletion of WRNIP1 increases the frequency of chromosome breaks upon PhenDC3 treatment**.
(a) Quantification of γH2AX intensity in shCtrl and shWRNIP1 carrying HeLa cells. Where indicated, cells were treated with G4 ligand PhenDC3 (10 μM) or Pyridostatin (10 μM). Error bars represent SEM of 3 biological replicates on graph. p-values were obtained by ANOVA (Origin Pro) (*P<0.05, **P<0.01, and ***P<0.001). Statistical data is provided in Table S4. **(b)**

Representative images of untreated and PhenDC3 or Pyridostatin treated (8 hours) cells from (a). Scale bars, 50 μm.

Supplementary Figure 5. **Original membranes of the Western blots**
(a) Original membrane of the experiment presented on Figure 2. **(b)** Original membrane of the experiment presented on Figure 5. **(c)** Original membrane of the experiment presented on Supplementary Figure 2.

Supplementary table 1: Sequence and labelling of oligonucleotides used in EMSA and fluorescence anisotropy experiments. G4-forming guanines are marked with bold.

Supplementary table 2: Sequences of oligonucleotides used for gene silencing

Supplementary table 3: List of antibodies used in Western blot and immunofluorescence experiments.

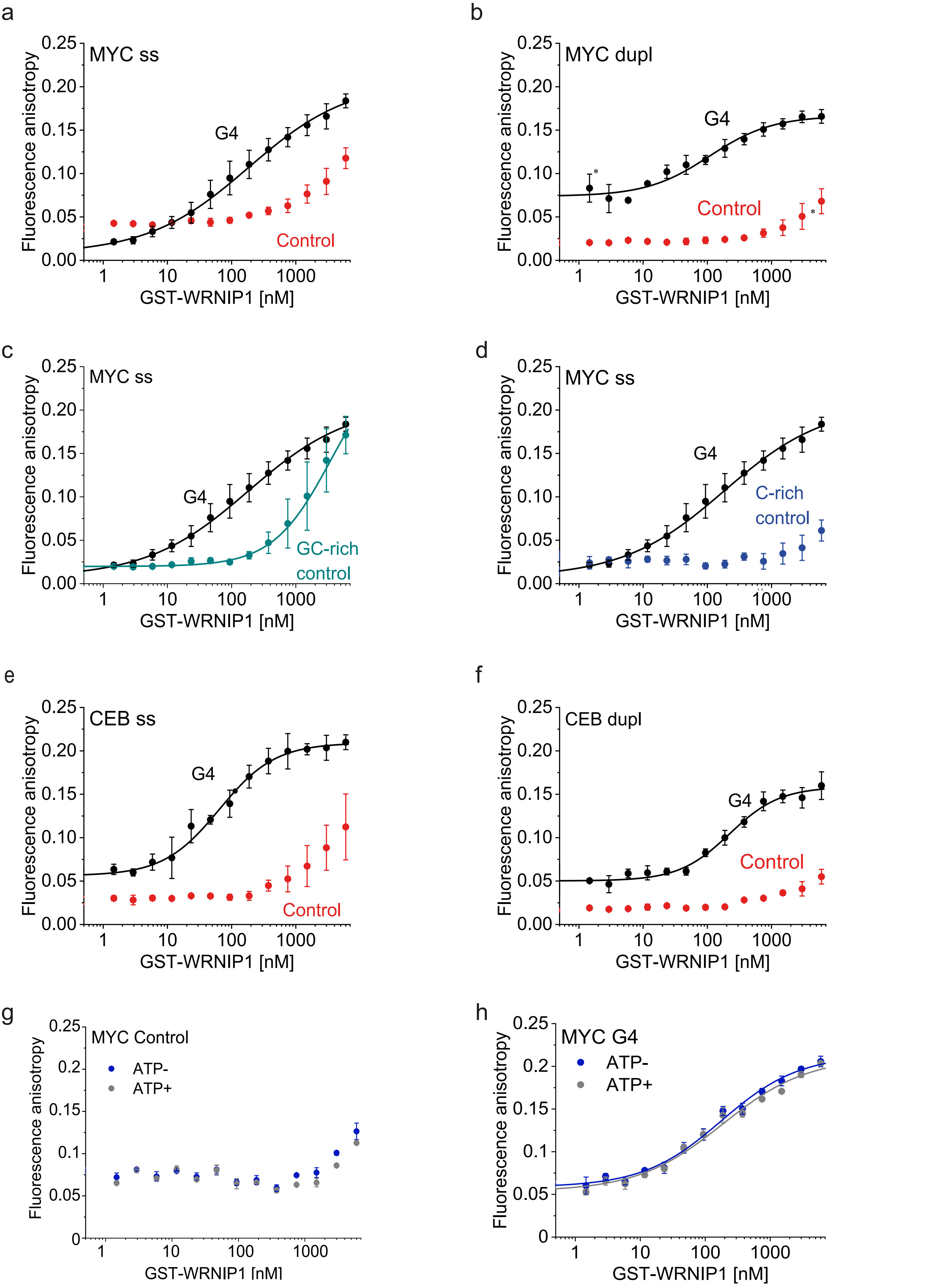

Supplementary Figure 1

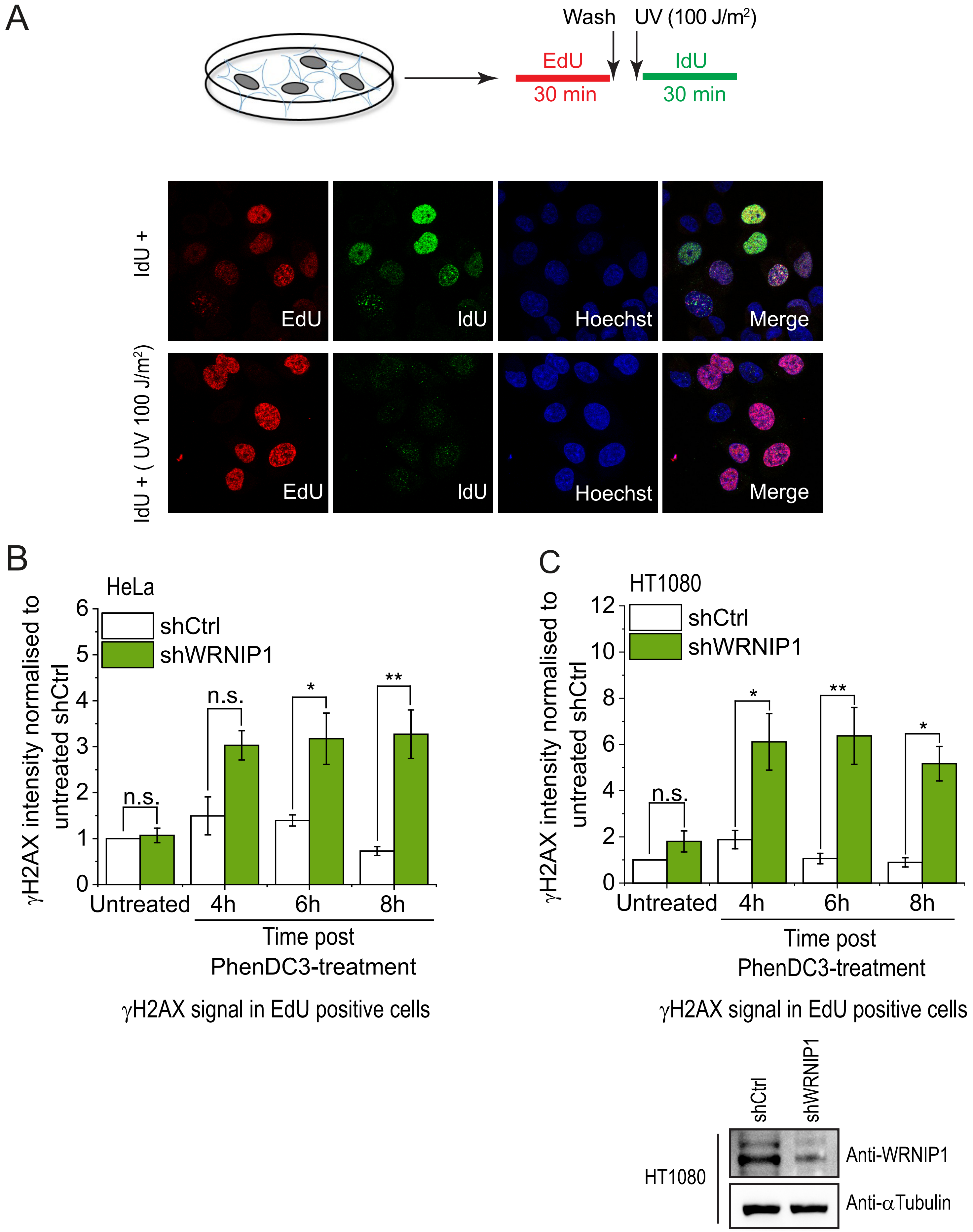

Supplementary Figure 2

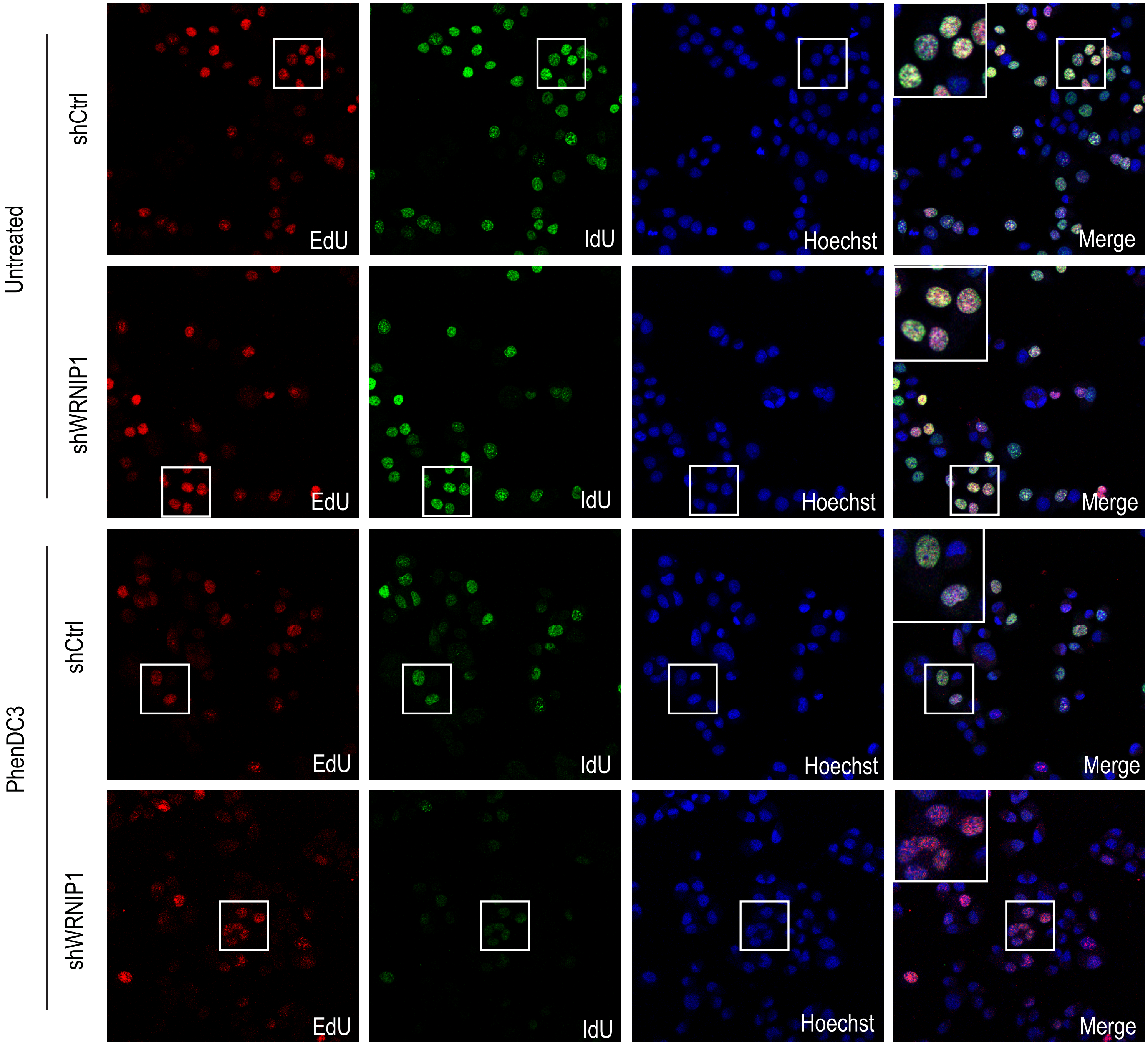

Supplementary Figure 3

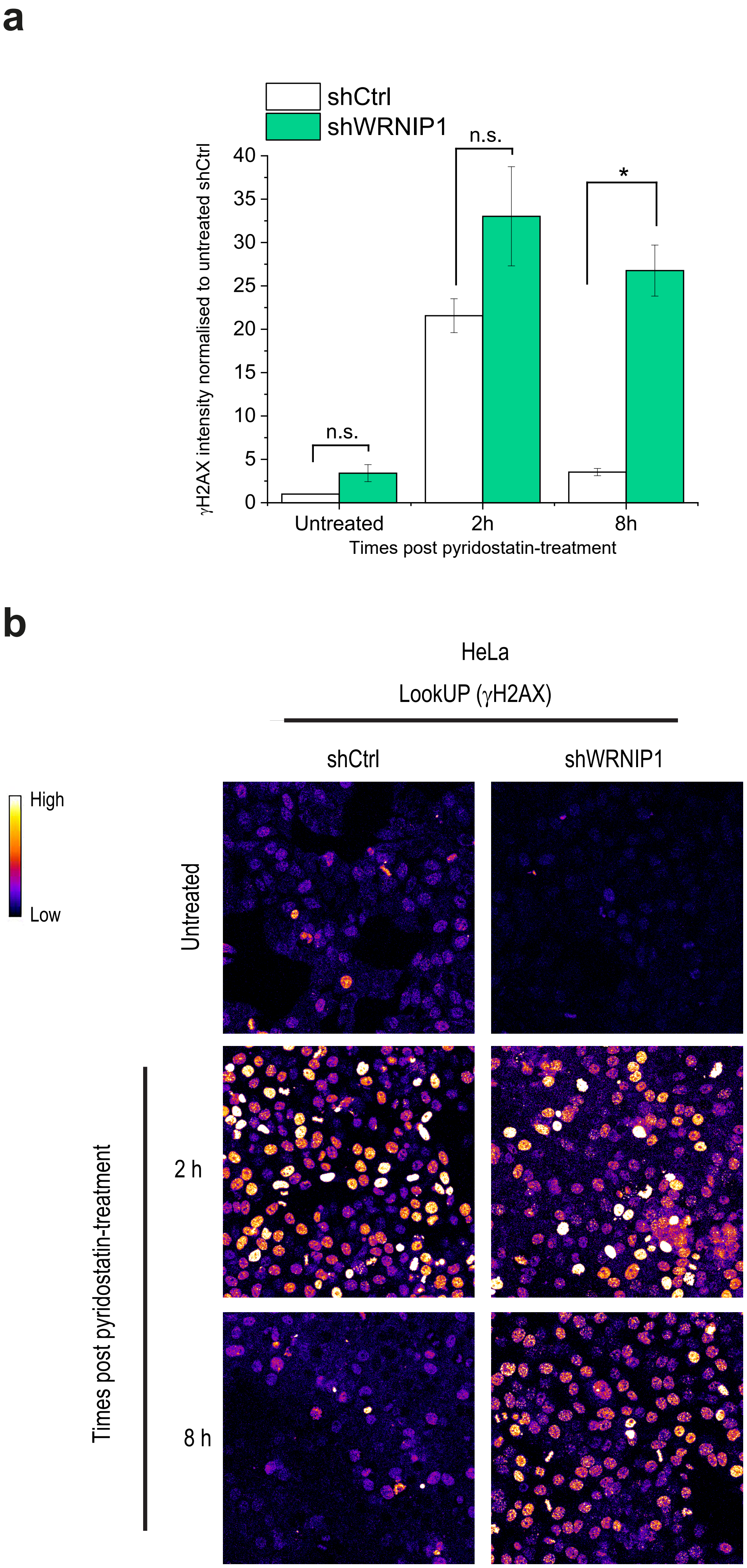

Supplementary Figure 4

a

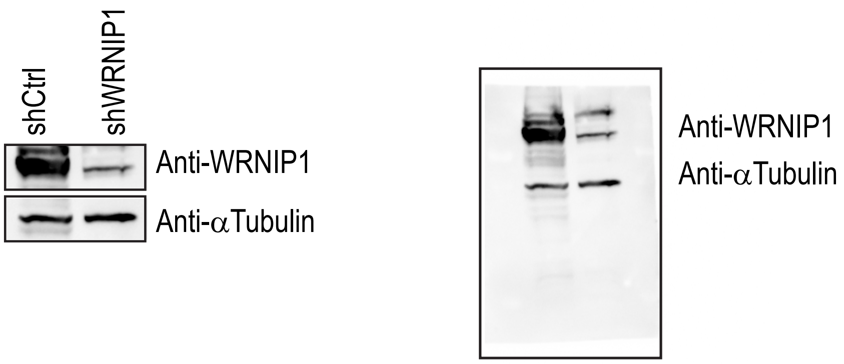

b

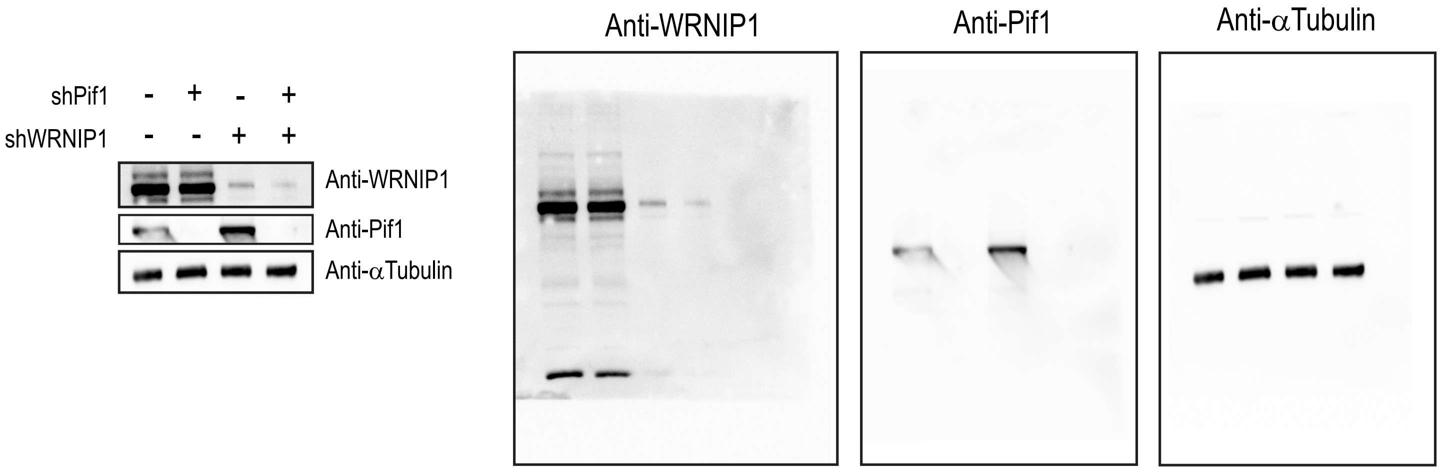

c

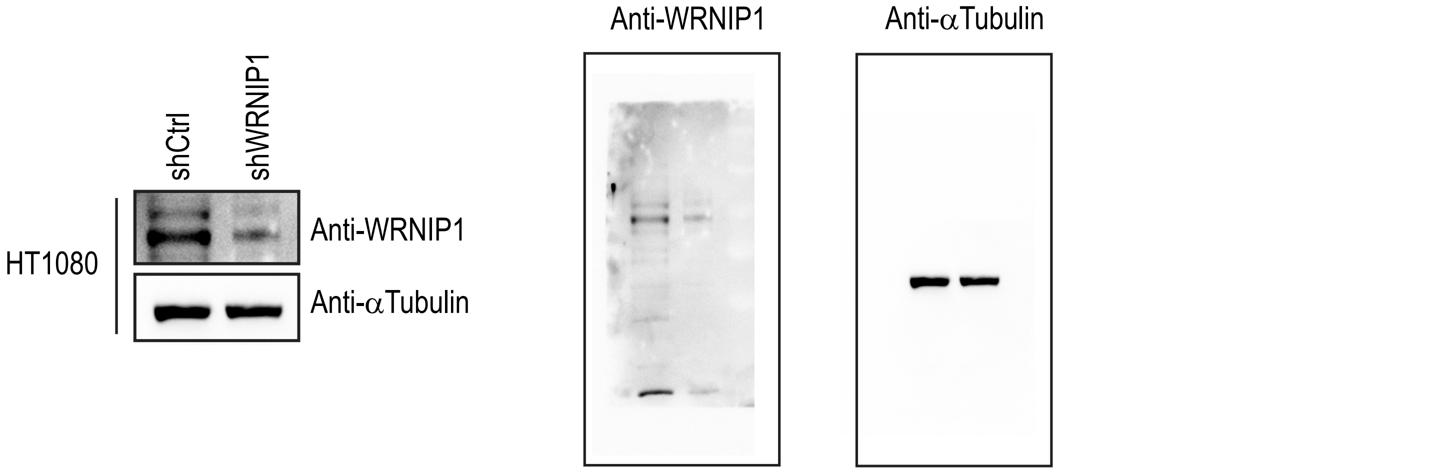

Supplementary Figure 5

**Name**	**Type**	**Sequence (5'-3')**	**Label**
MYC (Myc2345)	G4	TGA**GGG**T**GGG**TA**GGG**T**GGG**TGCGTCTG CGGCTGGCTCGAGGC	5’ FITC
	Control	GTGAGATGTTGACCATGGGTGCGTCTGC GGCTGGCTCGAGGC	5’ FITC or 5’ Cy3
	GC-rich control	TGAGTGTGAGTGGTGTGAGAGCGGCGG CGGCTGGCGCGAGGC	5’ FITC
	C-rich control	ACTCCCACCCATCCCACCCACGCAGAC GCCGACCGAGCTCCG	5’ FITC
CEB (CEB25)	G4	AA**GGG**T**GGG**TGTAAGTGT**GGG**T**GGG**T GCGTCTGCGGCTGGCTCGAGGC	5’ FITC
	Control	AAGCCTTATCTGTAAGTGTATGTCAAT GCGTCTGCGGCTGGCTCGAGGC	5’ FITC or 5’ Cy3
Complementary strand		TTTGCCTCGAGCCAGCCGCAGACG	-

Supplementary table 1

Name	Sequence	ID of oligo	ID (in pRNAU6-NEO)
shControl	GATCCCCTTCTCCGAACGTGTCACGTTTCAAGA GAACGTGACACGTTCGGAGAATTTTTGGAA	PO2607	PBP208
	AGCTTTTCCAAAAATTCTCCGAACGTGTCACGT TCTCTTGAAACGTGACACGTTCGGAGAAGGG	PO2608	
shWRNIP1	GATCTGGAGATCCGACAGATGCTATTCAAGAG ATAGCATCTGTCGGATCTCCTCTTTTTA	O2791	PBP398
	AGCTTAAAAAGAGGAGATCCGACAGATGCTAT CTCTTGAATAGCATCTGTCGGATCTCCA	O2792	
shPif1a	GATCTGGCCAGAGCATCTTCTTCATTCAAGAGA AATGAAGAAGAATGCTCTGGCCTTTTTA	PO499	PBP427
	AGCTTAAAAAAGGCCAGAGCATTCTTCTTCATT TCTCTTGAATGAAGAAGATGCTCTGGCCA	PO500	
	AGCTTAAAAAAATTCCCACTACTTTGCAAGTAAT CTCTTGAATTACTTGCAAAGTAGTGGGA	PO478	
	AGCTTAAAAAATTGGTGGTTACCTGCAACTATTC TCTTGAATTTACTCCAATTGCTACAGA	O3817	
	AGCTTAAAAACTAGAAATCTGTTGCTATGTCTC TTGCATAGCAACAGATTTCTAGA	O4172	

Supplementary table 2

Name	Host	Company	Catalogue Number	Dilution
Anti-WRNIP1	Rabbit	Abcam	ab99316	WB_1:2000
Anti-Pif1	Mouse	Santa cruz	sc-48377	WB_1:2000
Anti-DNA G-quadruplex (clone BG4)	*E. coli*	Millipore	MABE917	IF_1:200
Anti-FLAG	Mouse	Sigma	F1804	WB_1:1000; IF_1:200
Anti-γH2AX	Rabbit	Abcam	ab81299	IF_1:1000
Anti-IdU	Rat	Abcam	ab187742	IF_1:100
Anti-Tubulin	Mouse	Thermo Fisher	sc-62204	WB_1:2000
Anti-rabbit IgG Alexa Fluor 488	Goat	Invitrogen	A11008	IF_1:1000
Anti-mouse IgG Alexa Fluor 555	Goat	Invitrogen	A21422	IF_1:1000
Anti-rabbit IgG Alexa Fluor 555	Goat	Invitrogen	A21428	IF_1:1000
Anti-rat Alexa IgG Fluor 488	Goat	Invitrogen	A11006	IF_1:1000
Anti-mouse HRP	Goat	Invitrogen	62-6520	WB_1:3000
Anti-rabbit HRP	Swine	Dako	P0399	WB_1:3000

Supplementary table 3
